# Supplementary material for: Mapping open knowledge institutions: an exploratory analysis of Australian universities
Source: PeerJ. 2021 May 11;9:e11391. doi: 10.7717/peerj.11391 (PMC8121066; doi:10.7717/peerj.11391)

**Supplementary information**

**Appendix A: Justification for the “event_total” definition**

To be consistent with the construction of other indicators relating to research outputs, we have selected to base the “event_total” indicator on counting the number of publications satisfying the condition of having at least one Crossref event. However, the choice of “at least one” needs some justification. Hence, we take a closer look at how the universities’ performances change when we adjust the condition of “at least one” to “at least 2”, “at least 3”, etc. The results are summarised in Figure A1.

**Figure A1: Proportion of outputs with specified minimum numbers of Crossref events, for all 43 universities.**


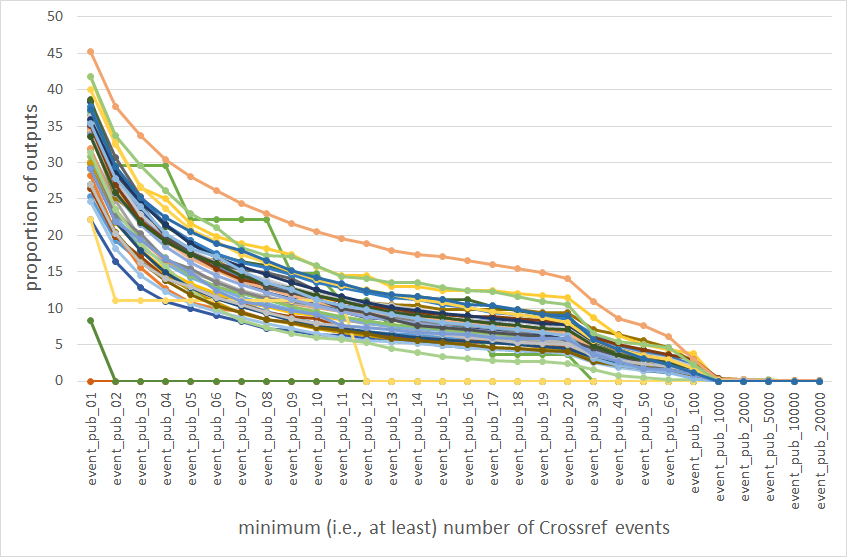


Figure A1 records, for each university (represented by each line), the proportion of its outputs meeting the conditions of having at least some number of Crossref events. We observe exponential decreases in the proportions as the requirement for minimum number of Crossref events increases. This is not unexpected given the exponential increase in number of events when outputs are arranged in order of number of events, i.e., there is a large number of outputs with 0 events, followed by one event, and so on, with very few number of outputs associated with extremely high numbers of events.

We note that there exist a few large jumps. These are representative of universities with very small numbers of outputs. Apart from these, the decreases in proportions of outputs generally appears to be in parallel. Hence, the relative performance (i.e., ranks) across the universities does not appear to drastically change due to changes to the required minimum number of events. As a result, we use “at least one” for simplicity.

We have also avoided using the actual value of event counts at the output level for constructing this indicator, such as the average number of events per article. Such a measure is highly influenced by outliers. For example, a small university in our data has one publication with more than 10000 tweets, significantly higher than the rest of its outputs. This publication includes more than 15000 signatories from other scientists, which may have had an impact on the number of tweets. This single output resulted in the university to have a much higher average event count than all other universities, albeit only having a fraction of number of output compared to other highly ranked universities.

This is an example of the general outlier problem for such data. If such multiplicative nature is persistent in the data, then an alternative measure of central tendency may be appropriate, e.g., geometric mean. But the extent to which such an approach can alleviate the effects of these observations is yet unknown. Hence, we have decided not to aggregate the number of events due to this issue.

**Appendix B: Data collection on policies and infrastructure**

We gathered documents from university public websites supplemented by directories and collections such as the Directory of Open Access Repositories (DOAR) (<http://v2.sherpa.ac.uk/opendoar/>), the Registry of Open Access Repository Mandates and Policies (ROARMAP) (<https://roarmap.eprints.org/>) and Politicas MELIBEA (<https://www.accesoabierto.net/politicas/>), a directory and estimator of OA policies for institutional repositories and practices. We developed a user-assisted tool to automate the search, retrieval and downloading of library access policy, OA policy and diversity policy documents from university websites. The tool consists of a Jupyter notebook supported by a small library of Python code. Using the Bing search engine API it executes a search against the URL for a specific university website recorded in the Global Research Identifier Database - GRID (<https://www.grid.ac/>). The code and an example Jupyter notebook are available at <https://doi.org/10.5281/zenodo.1438874>. This process was supplemented with human search and retrieval where necessary.

Subsequently, information on policies surrounding library access, OA and diversity were manually retrieved and used to answer a number of Yes/No questions (1=Yes; 0=No). These are then used to construct respective indicators in the following way:

- Library public access score (“policy_lib”, score out of 3):
- Is the library accessible by the public?
- Is the library freely (i.e., no fee) accessible by the public?
- Is the library accessible without restrictions (e.g., ID requirements)?
- OA score (“policy_div”, score out of 5):
- Does the university have an OA policy or statement?
- Does the university provide extra funding for OA publishing?
- Does the university have an OA repository?
- Does the university have a data sharing policy or statement?
- Does the university have an open data repository?
- Diversity policy score (“policy_div”, score out of 2):
- Does the university have a policy on employment equity, equality or equity?
- Does the university have a policy on staff diversity?

The process of retrieving policy documents and related information involved a vast amount of manual work, including several reviews of documents, manual searches and examining multiple weblinks. This process is described in detail in Wilson et al. (2019a, 2019b, 2020). The policy documents examined are copyright as per the respective universities and are used here for study and research only.

**Appendix C: Data collection and analysis of annual reports**

The individual annual reports of the universities are manually downloaded (if publicly available). A Python script is used to convert these PDF files into text file format, convert words in the documents into tokens, and identify a set of predefined phrases in each text document and record the number of times each phrase appears in the documents. The phrases are grouped into the three platforms of diversity, communication and coordination, with the aggregated relative frequency (out of total number of words in the document) used for the respective OKI indicators.

The downloaded annual reports and python scripts used for text analysis are made available on Zenodo (<https://doi.org/10.5281/zenodo.4034821>). The words used and the groupings can be found in the JSON file “words.json”, with the respective final word counts and relative frequencies recorded in “AU_2017.csv”.

The annual report PDF files are copyright as per the respective universities, where applicable. They are used here for study and research purposes only.

**Appendix D: Additional outputs for descriptive analysis**

Table D1 provides a number of summary statistics for each of the indicators, along with the number of missing values within each indicator.

**Table D1: Summary statistics of indicators.**

|  | **Min** | **1st Qu** | **Mean** | **Median** | **3rd Qu** | **Max** | **NA** |
| --- | --- | --- | --- | --- | --- | --- | --- |
| **oa_total** | 25.00 | 38.82 | 43.88 | 43.37 | 48.60 | 66.67 | 0 |
| **oa_gold** | 8.33 | 18.65 | 22.01 | 20.77 | 22.67 | 66.67 | 0 |
| **oa_bronze** | 0.00 | 6.93 | 7.89 | 8.33 | 9.66 | 12.04 | 0 |
| **oa_green** | 25.00 | 32.73 | 36.79 | 36.44 | 40.90 | 52.75 | 0 |
| **oa_green_only** | 0.00 | 11.27 | 13.98 | 12.40 | 17.00 | 27.64 | 0 |
| **output_div** | 0.00 | 0.17 | 0.23 | 0.22 | 0.27 | 0.44 | 0 |
| **collab_total** | 41.67 | 72.39 | 75.23 | 74.61 | 77.79 | 100.00 | 0 |
| **collab_aus** | 26.74 | 39.67 | 48.19 | 45.13 | 50.62 | 100.00 | 0 |
| **collab_other** | 0.00 | 51.15 | 53.24 | 55.87 | 59.34 | 70.67 | 0 |
| **collab_ind** | 2.30 | 3.20 | 3.87 | 4.00 | 4.40 | 5.30 | 17 |
| **event_total** | 0.00 | 29.18 | 31.65 | 33.62 | 36.38 | 45.27 | 0 |
| **walk_score** | 7.00 | 45.50 | 62.77 | 61.00 | 87.00 | 100.00 | 0 |
| **web_score** | 21.00 | 37.25 | 40.71 | 43.00 | 46.00 | 51.00 | 1 |
| **indigenous** | 0.00 | 0.67 | 1.60 | 1.12 | 1.66 | 16.13 | 0 |
| **women_above_sl** | 26.03 | 30.83 | 35.30 | 33.10 | 37.69 | 100.00 | 1 |
| **women_sl** | 13.33 | 43.75 | 47.54 | 46.30 | 50.56 | 100.00 | 2 |
| **women_l** | 42.11 | 52.11 | 55.70 | 54.89 | 60.65 | 67.57 | 2 |
| **women_below_l** | 44.13 | 48.71 | 56.67 | 55.56 | 61.75 | 100.00 | 3 |
| **women_acad** | 35.68 | 43.64 | 47.26 | 47.04 | 51.40 | 60.61 | 4 |
| **women_non_acad** | 53.85 | 65.00 | 67.40 | 66.77 | 68.87 | 100.00 | 1 |
| **policy_lib** | 0.00 | 2.00 | 2.15 | 2.00 | 2.25 | 3.00 | 0 |
| **policy_oa** | 1.00 | 3.00 | 3.23 | 3.00 | 4.00 | 5.00 | 0 |
| **policy_div** | 0.00 | 2.00 | 1.74 | 2.00 | 2.00 | 2.00 | 0 |
| **ann_rep_diversity** | 0.0017 | 0.0027 | 0.0034 | 0.0033 | 0.0040 | 0.0080 | 9 |
| **ann_rep_comm** | 0.0002 | 0.0004 | 0.0007 | 0.0006 | 0.0008 | 0.0024 | 9 |
| **ann_rep_coord** | 0.0028 | 0.0045 | 0.0052 | 0.0053 | 0.0058 | 0.0069 | 9 |
| **total_rev** | 40742.00 | 380714.50 | 821233.10 | 664774.00 | 961264.50 | 2501975.00 | 4 |

To highlight the extreme points for each OKI indicator and to allow for cross-comparison of these indicators, we normalise each indicator using the min-max rescaling. This results in each indicator being rescaled to the range between 0 and 100. The boxplots of these normalised indicators are presented in Figure D1. The dots represent extreme observations.

**Figure D1: Boxplots of normalised observations for OKI indicators.**


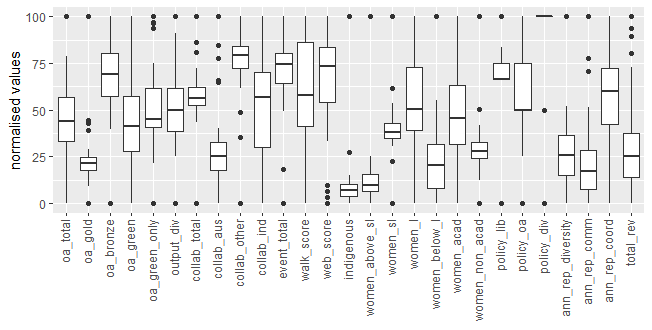


**Appendix E: Additional PCA and cluster analysis results**

**Figure E1: Percentage of variance explained by PCs (left) and Scree plot of eigenvalues with Kaiser Criterion (right), for ROBPCA.**


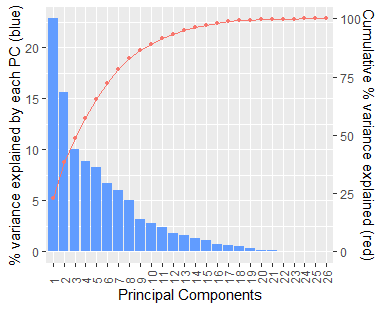

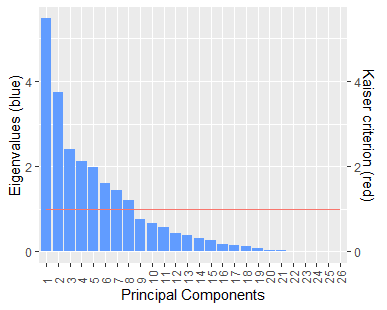


**Table E1: Standardised loadings on the first 8 PCs from the Spearman PCA.**

| **Indicators** | **PC1** | **PC2** | **PC3** | **PC4** | **PC5** | **PC6** | **PC7** | **PC8** |
| --- | --- | --- | --- | --- | --- | --- | --- | --- |
| **oa_total** | -0.06 | 0.38 | -0.01 | 0.24 | 0.22 | -0.01 | 0.14 | -0.02 |
| **oa_gold** | 0.12 | 0.37 | -0.05 | -0.02 | -0.18 | 0.08 | -0.16 | 0.00 |
| **oa_bronze** | -0.21 | 0.14 | 0.09 | -0.32 | 0.00 | 0.28 | 0.12 | 0.03 |
| **oa_green** | -0.11 | 0.39 | -0.03 | 0.24 | 0.18 | -0.10 | 0.16 | 0.08 |
| **oa_green_only** | -0.17 | 0.21 | -0.01 | 0.24 | 0.26 | -0.22 | 0.30 | 0.16 |
| **output_div** | 0.01 | -0.36 | 0.04 | 0.17 | 0.11 | -0.18 | 0.12 | -0.02 |
| **collab_total** | 0.06 | 0.33 | -0.29 | -0.09 | 0.02 | -0.11 | -0.21 | -0.18 |
| **collab_aus** | 0.31 | 0.10 | -0.07 | -0.13 | -0.05 | -0.04 | 0.01 | 0.07 |
| **collab_other** | -0.23 | 0.28 | -0.11 | -0.11 | 0.04 | 0.06 | -0.16 | -0.22 |
| **collab_ind** | -0.20 | 0.11 | 0.04 | 0.08 | -0.32 | 0.14 | 0.42 | -0.22 |
| **event_total** | 0.09 | 0.27 | 0.35 | -0.10 | -0.17 | -0.10 | 0.06 | 0.26 |
| **walk_score** | -0.21 | 0.04 | -0.33 | -0.34 | 0.10 | -0.01 | -0.07 | -0.12 |
| **web_score** | -0.02 | -0.01 | 0.24 | -0.34 | 0.20 | -0.03 | 0.22 | 0.45 |
| **indigenous** | 0.17 | 0.02 | 0.20 | 0.45 | 0.02 | 0.07 | -0.08 | -0.28 |
| **women_above_sl** | 0.32 | 0.00 | -0.04 | -0.16 | 0.12 | -0.14 | -0.06 | -0.03 |
| **women_sl** | 0.31 | 0.13 | -0.02 | -0.09 | 0.05 | -0.19 | -0.01 | -0.08 |
| **women_l** | 0.33 | 0.13 | 0.08 | 0.03 | 0.10 | -0.06 | 0.00 | 0.05 |
| **women_below_l** | 0.29 | 0.05 | -0.03 | 0.04 | -0.18 | 0.11 | 0.23 | 0.04 |
| **women_acad** | 0.36 | 0.09 | 0.03 | -0.04 | -0.01 | 0.00 | 0.08 | -0.03 |
| **women_non_acad** | 0.23 | 0.01 | 0.00 | -0.12 | 0.11 | 0.09 | 0.07 | -0.28 |
| **policy_lib** | 0.10 | -0.03 | 0.11 | 0.16 | 0.20 | 0.66 | 0.01 | -0.07 |
| **policy_oa** | -0.10 | 0.19 | 0.46 | -0.08 | -0.18 | 0.09 | -0.24 | 0.02 |
| **policy_div** | -0.08 | 0.01 | 0.46 | -0.13 | 0.26 | 0.00 | -0.30 | -0.22 |
| **ann_rep_diversity** | -0.11 | -0.04 | 0.29 | -0.04 | 0.16 | -0.45 | 0.03 | -0.36 |
| **ann_rep_comm** | 0.05 | -0.01 | -0.11 | 0.02 | 0.60 | 0.21 | -0.13 | 0.18 |
| **ann_rep_coord** | 0.08 | -0.01 | 0.06 | -0.33 | 0.17 | 0.08 | 0.52 | -0.40 |

**Table E2: Standardised loadings on the first 7 PCs from ROBPCA.**

| **Indicators** | **PC1** | **PC2** | **PC3** | **PC4** | **PC5** | **PC6** | **PC7** |
| --- | --- | --- | --- | --- | --- | --- | --- |
| **oa_total** | -0.15 | -0.41 | 0.03 | 0.04 | 0.03 | 0.13 | 0.13 |
| **oa_gold** | -0.21 | -0.13 | -0.04 | -0.01 | -0.05 | -0.02 | -0.04 |
| **oa_bronze** | 0.00 | -0.18 | 0.00 | 0.20 | -0.35 | -0.21 | -0.13 |
| **oa_green** | -0.13 | -0.48 | 0.03 | -0.01 | 0.14 | 0.21 | 0.18 |
| **oa_green_only** | 0.10 | -0.25 | 0.10 | -0.03 | 0.27 | 0.29 | 0.28 |
| **output_div** | 0.34 | 0.27 | 0.00 | -0.11 | 0.09 | 0.24 | 0.05 |
| **collab_total** | -0.27 | -0.09 | 0.18 | 0.00 | -0.06 | -0.09 | -0.12 |
| **collab_aus** | -0.39 | 0.14 | 0.14 | -0.03 | 0.03 | 0.04 | -0.09 |
| **collab_other** | -0.04 | -0.24 | 0.17 | 0.07 | -0.09 | -0.16 | -0.10 |
| **collab_ind** | 0.13 | -0.23 | 0.09 | 0.03 | -0.31 | 0.11 | 0.34 |
| **event_total** | -0.32 | -0.08 | -0.21 | -0.07 | -0.11 | 0.03 | -0.01 |
| **walk_score** | 0.07 | -0.12 | 0.28 | 0.40 | -0.27 | -0.08 | -0.24 |
| **web_score** | 0.11 | 0.09 | -0.53 | 0.31 | -0.32 | -0.10 | 0.46 |
| **indigenous** | -0.05 | 0.02 | -0.02 | -0.10 | 0.10 | 0.04 | -0.04 |
| **women_above_sl** | -0.20 | 0.14 | -0.02 | 0.02 | -0.05 | 0.01 | -0.04 |
| **women_sl** | -0.27 | 0.08 | -0.07 | -0.05 | -0.12 | 0.10 | 0.02 |
| **women_l** | -0.36 | 0.09 | -0.17 | -0.02 | 0.03 | 0.08 | 0.04 |
| **women_below_l** | -0.21 | 0.17 | -0.04 | 0.05 | 0.00 | 0.15 | 0.21 |
| **women_acad** | -0.29 | 0.17 | -0.10 | -0.01 | -0.06 | 0.08 | 0.05 |
| **women_non_acad** | -0.08 | 0.08 | 0.03 | 0.06 | -0.02 | 0.05 | -0.06 |
| **policy_lib** | -0.03 | 0.12 | 0.03 | 0.19 | 0.18 | -0.17 | -0.02 |
| **policy_oa** | -0.02 | -0.28 | -0.40 | -0.31 | -0.11 | -0.11 | -0.13 |
| **policy_div** | 0.17 | -0.12 | -0.41 | -0.12 | -0.02 | -0.04 | -0.42 |
| **ann_rep_diversity** | 0.09 | -0.11 | -0.16 | -0.18 | 0.04 | 0.34 | -0.30 |
| **ann_rep_comm** | -0.06 | -0.12 | -0.30 | 0.60 | 0.56 | -0.05 | -0.12 |
| **ann_rep_coord** | 0.02 | 0.07 | -0.03 | 0.33 | -0.28 | 0.69 | -0.27 |

**Table E3: Rotated loadings on first 3 PCs from Spearman PCA and ROBPCA.**

|  | **Spearman PCA** | | | **ROBPCA** | | |
| --- | --- | --- | --- | --- | --- | --- |
| **Indicators** | **PC1** | **PC2** | **PC3** | **PC1** | **PC2** | **PC3** |
| **oa_total** | 0.0109 | **0.3864** | 0.0238 | 0.0065 | **-0.4351** | -0.0563 |
| **oa_gold** | 0.1902 | **0.3421** | -0.0243 | -0.1602 | -0.1953 | -0.0386 |
| **oa_bronze** | -0.1717 | 0.1703 | 0.1055 | 0.0602 | -0.1625 | -0.0445 |
| **oa_green** | -0.0339 | **0.4057** | 0.0049 | 0.0521 | **-0.4936** | -0.0795 |
| **oa_green_only** | -0.1297 | 0.2425 | 0.0154 | 0.2053 | **-0.2073** | 0.0027 |
| **output_div** | -0.0635 | **-0.3589** | 0.0089 | 0.2167 | 0.376 | 0.0145 |
| **collab_total** | 0.1128 | **0.3403** | -0.2672 | -0.1656 | **-0.2224** | 0.191 |
| **collab_aus** | **0.3225** | 0.0486 | -0.0725 | **-0.3655** | -0.0514 | 0.2436 |
| **collab_other** | -0.1727 | **0.3294** | -0.081 | 0.0898 | **-0.2659** | 0.0974 |
| **collab_ind** | -0.1689 | 0.1459 | 0.0541 | 0.2206 | -0.1703 | 0.0006 |
| **event_total** | 0.1528 | 0.2146 | **0.3684** | **-0.3148** | -0.1583 | -0.1703 |
| **walk_score** | -0.2091 | 0.1048 | **-0.3175** | 0.1823 | -0.1358 | 0.2174 |
| **web_score** | -0.0136 | -0.0255 | 0.2427 | -0.0768 | 0.2265 | **-0.4968** |
| **indigenous** | 0.178 | -0.0342 | 0.1933 | -0.0574 | 0.0007 | -0.0086 |
| **women_above_sl** | **0.3146** | -0.0625 | -0.0573 | -0.2317 | 0.0551 | 0.0511 |
| **women_sl** | **0.3234** | 0.0659 | -0.0216 | **-0.2917** | -0.0169 | 0.0082 |
| **women_l** | **0.3489** | 0.0529 | 0.0754 | **-0.4042** | -0.0272 | -0.071 |
| **women_below_l** | **0.2949** | -0.0027 | -0.0354 | **-0.2519** | 0.0803 | 0.0481 |
| **women_acad** | **0.3737** | 0.0147 | 0.0195 | **-0.3454** | 0.0564 | 0.0012 |
| **women_non_acad** | 0.2253 | -0.0375 | -0.0142 | -0.0916 | 0.0305 | 0.0676 |
| **policy_lib** | 0.0995 | -0.0576 | 0.1069 | -0.0628 | 0.0903 | 0.0687 |
| **policy_oa** | -0.0523 | 0.1632 | **0.4806** | -0.028 | -0.186 | **-0.4547** |
| **policy_div** | -0.0614 | -0.0173 | **0.4627** | 0.0867 | 0.0374 | **-0.449** |
| **ann_rep_diversity** | -0.1093 | -0.0396 | **0.2901** | 0.0765 | -0.0358 | -0.1987 |
| **ann_rep_comm** | 0.0399 | -0.0081 | -0.1146 | -0.0895 | -0.0729 | **-0.3018** |
| **ann_rep_coord** | 0.0746 | -0.0321 | 0.058 | -0.0116 | 0.0784 | -0.0128 |

**Table E4: Proportion of PCs’ variances loaded by groups of OKI indicators.**

|  | **ROBPCA** | | |
| --- | --- | --- | --- |
| **Platforms** | **PC1** | **PC2** | **PC3** |
| **Diversity** | **66.3%** | 3.6% | 12.1% |
| **Communication** | 15.3% | **77.6%** | 5.7% |
| **Coordination** | 3.2% | 10.8% | **79.1%** |
| **“event_total”** | 9.9% | 2.5% | 2.9% |
| **“oa_bronze”** | 0.4% | 2.6% | 0.2% |
| **“collab_ind”** | 4.9% | 2.9% | 0.0% |

**Figure E2: Correlation circle against first 2 PCs using ROBPCA**


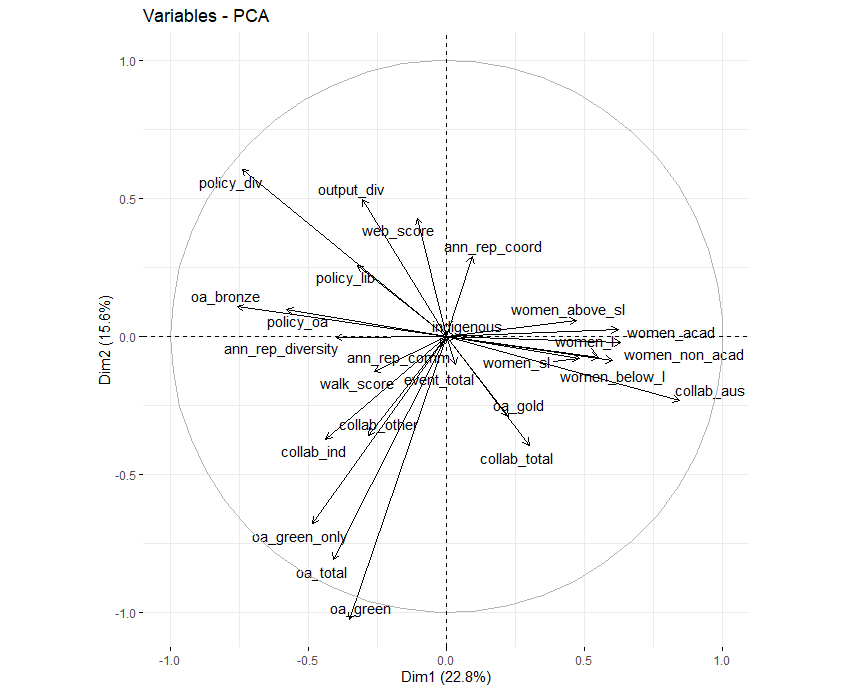


**Figure E3: Individual component plot for first two PCs from ROBPCA, with universities coloured by state.**


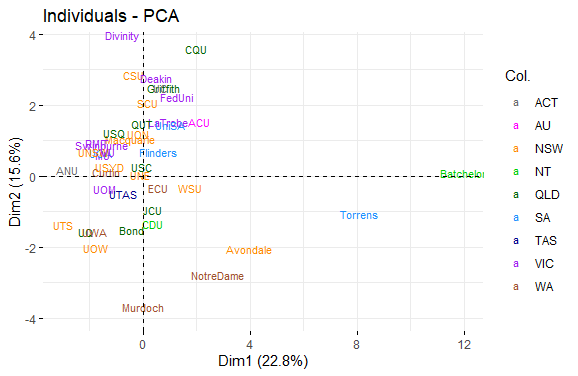


**Figure E4: Individual component plot for first two PCs from ROBPCA, with universities coloured by university network.**


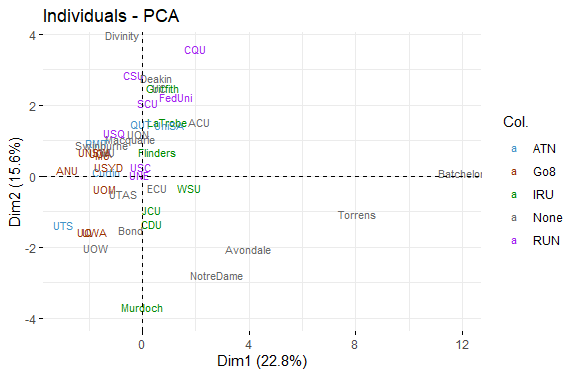


**Figure E5:  Dendrogram of hierarchical clustering of universities using ranks in OKI indicators, with universities coloured by state.**


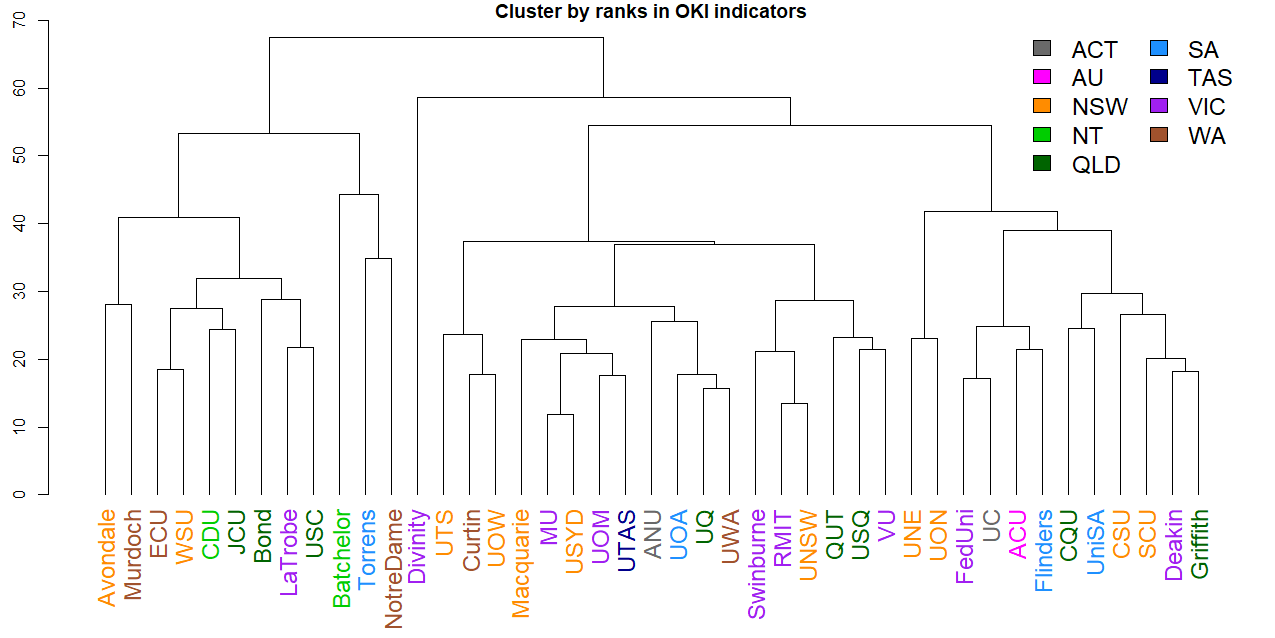


**Figure E6:  Dendrogram of hierarchical clustering of universities using OKI indicators, with universities coloured by state.**


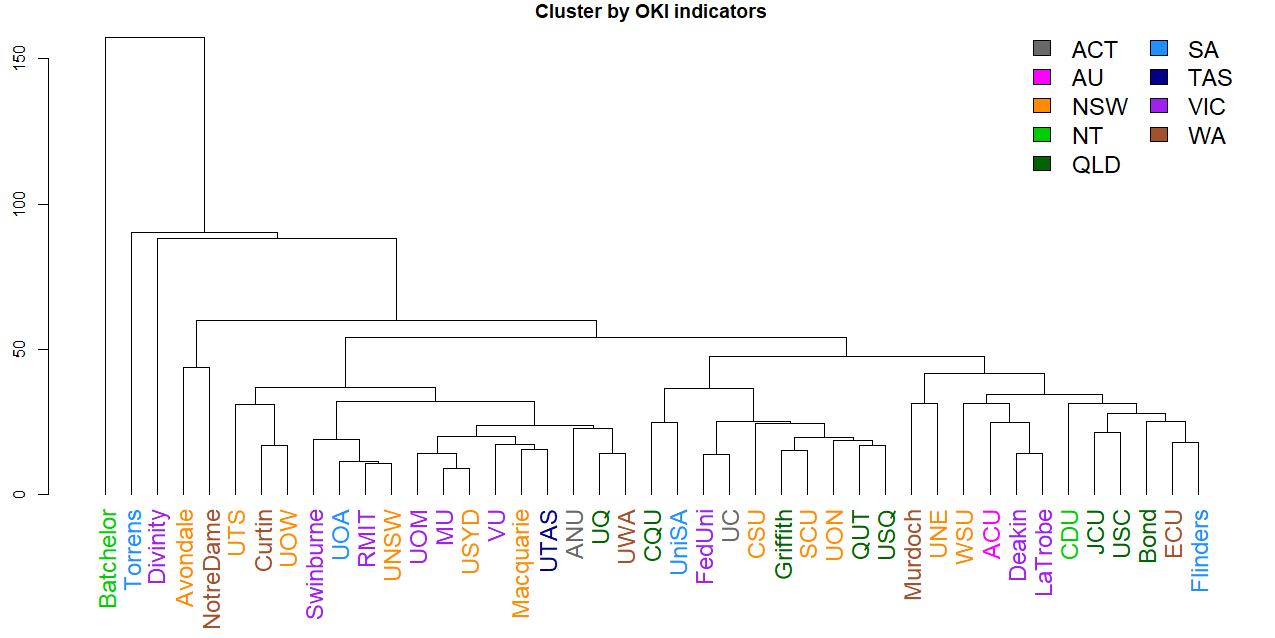

Supplement: Supplemental Information 1 [file peerj-09-11391-s001.docx]
